# Landscape connectivity for bobcat (*Lynx rufus*) and lynx (*Lynx canadensis*) in the Northeastern United States

**DOI:** 10.1371/journal.pone.0194243

**Published:** 2018-03-28

**Authors:** Laura E. Farrell, Daniel M. Levy, Therese Donovan, Ruth Mickey, Alan Howard, Jennifer Vashon, Mark Freeman, Kim Royar, C. William Kilpatrick

**Affiliations:** 1 Department of Biology, University of Vermont, Burlington, Vermont, United States of America; 2 Rubenstein School of Environment and Natural Resources, University of Vermont, Burlington, Vermont, United States of America; 3 U.S. Geological Survey, Vermont Cooperative Fish and Wildlife Research Unit, Rubenstein School of Environment and Natural Resources, University of Vermont, Burlington, Vermont, United States of America; 4 Department of Mathematics and Statistics, University of Vermont, Burlington, Vermont, United States of America; 5 Statistical Consulting Clinic, University of Vermont, Burlington, Vermont, United States of America; 6 Maine Department of Inland Fisheries and Wildlife, Bangor, Maine, United States of America; 7 Vermont Fish and Wildlife Department, Springfield, Vermont, United States of America; Smithsonian Conservation Biology Institute, UNITED STATES

## Abstract

Landscape connectivity is integral to the persistence of metapopulations of wide ranging carnivores and other terrestrial species. The objectives of this research were to investigate the landscape characteristics essential to use of areas by lynx and bobcats in northern New England, map a habitat availability model for each species, and explore connectivity across areas of the region likely to experience future development pressure. A Mahalanobis distance analysis was conducted on location data collected between 2005 and 2010 from 16 bobcats in western Vermont and 31 lynx in northern Maine to determine which variables were most consistent across all locations for each species using three scales based on average 1) local (15 minute) movement, 2) linear distance between daily locations, and 3) female home range size. The bobcat model providing the widest separation between used locations and random study area locations suggests that they cue into landscape features such as edge, availability of cover, and development density at different scales. The lynx model with the widest separation between random and used locations contained five variables including natural habitat, cover, and elevation—all at different scales. Shrub scrub habitat—where lynx’s preferred prey is most abundant—was represented at the daily distance moved scale. Cross validation indicated that outliers had little effect on models for either species. A habitat suitability value was calculated for each 30 m^2^ pixel across Vermont, New Hampshire, and Maine for each species and used to map connectivity between conserved lands within selected areas across the region. Projections of future landscape change illustrated potential impacts of anthropogenic development on areas lynx and bobcat may use, and indicated where connectivity for bobcats and lynx may be lost. These projections provided a guide for conservation of landscape permeability for lynx, bobcat, and species relying on similar habitats in the region.

## Introduction

Forest cover across the Northeastern United States has increased since the early nineteenth century as agricultural land has been abandoned. Populations of native carnivores have recovered in response to increased habitat. For example, a breeding population of lynx (*Lynx canadensis*) was absent from the region for decades but became reestablished naturally in Maine in 1999 [1], and more recently in New Hampshire [2], and Vermont [3]. Anthropogenic growth is now reversing the 150-year increase in habitat and is projected to continue, increasing habitat fragmentation and adding barriers to wildlife movement [4,5]. Long–term conservation of wide–ranging species depends on maintaining landscapes in which both local daily movements and longer inter-territorial dispersal events are possible [6,7]. Wide–ranging species such as bobcat (*Lynx rufus*) and lynx (*Lynx canadensis*) rely on connective habitat to facilitate seasonal range-shifts between heavily used core areas, as well as longer–distance movements such as dispersal that link subpopulations and permit gene flow [1, 8, 9]. Dispersal distances from natal ranges of up to 182 km for bobcat and 1100 km for lynx [10, 11], emphasize the value of regional-scale conservation strategies.

Setting such strategies presents three major challenges. First, species have differing habitat requirements that may require unique conservation solutions. Bobcats use broader landscape characteristics including natural habitats, riparian areas, edge, and natural cover in areas of moderate human use [12–14], but avoid areas of high human activity [15, 16]. Lynx are tightly tied to boreal forest and shrub scrub (shrub < 5 m tall > 20% of canopy), as these are favored habitat of their main prey, the snowshoe hare (*Lepus americanus*) [11]. Second, each species cues into certain habitats for food acquisition, denning, or travel at different scales [17–22]. The scale at which variables are examined can determine what scales are most important to utilization of resources, and influence the ability to accurately predict effects of habitat fragmentation on a particular species [23–25]. Thus, for effective conservation linkage strategies, the interactions of multiple habitat variables at a range of spatial scales should be investigated for each species [26–29]. And third, there is a need to map the amount and distribution of suitable habitat and the functional ability of the landscape to connect populations at a regional scale to aid decision making [30].

As change to the Northeastern landscape intensifies in order to accommodate a greater human population, maintaining ecosystem processes such as dispersal of wide ranging mammals depends on identifying networks of connective habitat linking high quality core areas. Efforts to create connectivity maps have been achieved for bobcats in New Hampshire and lynx in the Rocky Mountains, and sub home-range scales have been investigated for pumas, but corridor models using resource selection methods may not best predict landscape use [31–35]. Regional connectivity models where scaled variables are not modeler selected, but instead selected by distilling appropriate variables from locations, are lacking for lynx and bobcat in the Northeast. A pragmatic connectivity assessment will not only consider the spatial distribution of habitats across a landscape in the current time, but can also predict its effectiveness as landscapes change in the future. Identifying habitat and connective linkage areas, and determining how these may withstand development pressure, will help planners implement land use strategies that will facilitate both human and wildlife needs through landscape changes.

Here, we used a partitioned Mahalanobis Distance Squared analysis (*D*^2^) to derive a habitat suitability model for lynx and bobcat and map suitability across the Northeastern U.S. based on GPS location data and the spatial covariates that are associated with each location. The *D*^2^ approach is a Principal Components Analysis (PCA), but rather than focusing on components that explain the variation among locations, it reveals the habitat components that are most similar across all locations; these represent the minimum habitat requirements for each species and can be scored on a pixel-by-pixel basis [36]. These minimal habitat requirements are assumed to identify all usable areas, including movement and dispersal habitat. Our objectives were to: 1) use the *D*^2^ approach [36, 37] to identify the landscape characteristics, at biologically relevant spatial scales, that are consistently used by known populations of lynx and by bobcat in northern New England, and use the most highly validated model for each species to map a movement suitability value for each 30 m^2^ pixel across Vermont, New Hampshire and Maine; 2) use the *D*^*2*^ model for each species to identify current connectivity (linked areas conducive to movement) across subportions of the region by developing cost-distance based landscape conductivity maps—including an area recently recolonized by unstudied lynx, and 3) estimate the persistence of connectivity into the future in areas most likely to experience increased anthropogenic pressure.

## Methods

### Study area

**Fig 1:**
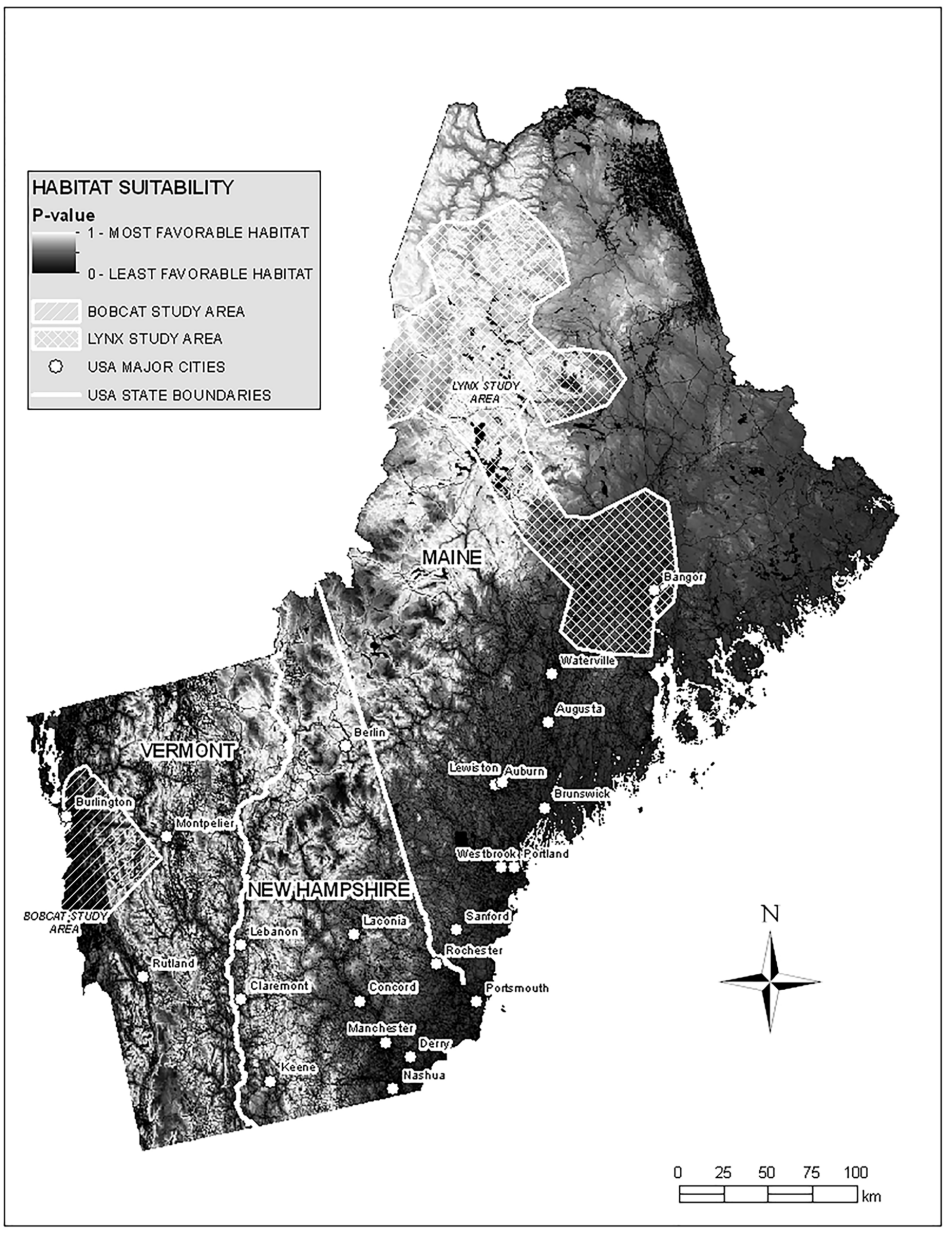
Regional area of analyses. Lynx habitat *P*–values mapped by 30 m^2^ pixel across Vermont, New Hampshire and Maine, with bobcat and lynx study areas indicated.

The regional area of analyses encompassed the states of Vermont (VT), New Hampshire (NH) and Maine (ME), an area of 132,313 km^2^ (Fig 1). Empirical data for bobcat were collected in northwestern Vermont, from the Champlain Valley lowlands eastward into the north central Green Mountains, with Lake Champlain forming the western boundary. Temperatures in the Champlain Valley are moderated by Lake Champlain; average daily highs range from over 21°C in July to -11°C in January. Summer temperatures in the mountains are often 6.7°C cooler [38]. Elevation ranged from 29 m at the edge of Lake Champlain to 1,245 m on top of Camel’s Hump Mountain [38]. The 2,997 km^2^ study area, including portions of Chittenden, Lamoille, Addison, and Washington Counties, contained a range of natural habitats interspersed with rural agricultural and more densely developed areas [39]. Lowland forests were predominantly hardwood; softwoods increased with elevation into the Green Mountains [38]. Road density in the bobcat study area ranged from 0 to between 0.84 linear km/ km^2^ and 9.36 km/ km^2^ [40], while human density in the Champlain Basin averaged 23.5 people/km^2^ [41].

Empirical lynx data were collected from a 19,875 km^2^ area, primarily in the Musquacook lakes region of northwestern Maine (Fig 1). Average daily highs ranged from 24 C in July to -7 in January [42]. Elevation ranged between 250 and 550 m over rolling hills and wide valleys. A matrix of spruce-fir forest was interspersed with lowland spruce-tamarack-cedar forests and hardwood dominated ridges. Much of the area was clear cut in the 1980s, leading to extensive areas of regenerating forest in the 1990s. Although road density is relatively high (~100km/100km^2^), these dirt roads were used primarily for wood extraction and most were not maintained. Human density was 0 in the majority of the lynx study area, as activity was limited to seasonal camps and logging operations [20]. One lynx (L114) made a long range movement into the area around Bangor, ME, where road densities of well-travelled roads are high, and human density exceeds 2,500/km^2^.

### Location data

Bobcat GPS radio–collar locations were provided by the Vermont Cooperative Fish and Wildlife Research Unit from a study conducted between 2005 and 2008 [39] (S1 Table). Lynx GPS radio–collar locations were collected by Maine Department of Inland Fisheries and Wildlife between 2005 and 2010 during a study that started in 1999 [20, 43]. Modeling was done from these data.

GPS radio–collar locations were taken at regular intervals 24 hours a day. The full range of locations were used to incorporate less frequently used habitats such as those used by individuals moving across longer distances. Locations from each cat were edited to 4 hours apart. We balanced available data per cat with activity patterns to maximize independence, time between locations and available data. There may be autocorrelation of some resting locations, though both species move throughout the day depending on climate and daylight hours, and consistency of use of certain habitats for both species underscores the importance of specific landscape variables. A maximum of 200 points were taken from each collar to minimize weighting of data (S2 and S3 Tables). It was assumed that telemetry error for the ATS G2004 (Advanced Telemetry Systems, Isanti MN), Lotek 3300S (Lotek Wireless Inc., Newmarket, Ontario) and Sirtrack^®^ (Hawkes Bay, New Zealand) collars used was absorbed within the 30 m pixels and the buffers around each location used in this analysis (T. Garin, Advanced Telemetry Systems, pers. com.) [44, 45].

Lynx were found to be recolonizing New Hampshire after modeling but during the mapping phase of this study, and we took the opportunity to explore how the lynx population may be recolonizing parts of its historic range. A connectivity map was produced using, instead of core habitat blocks, 142 uncollared lynx locations from 2006 through 2011, verified by the US Fish and Wildlife Service and state wildlife departments using photos, tracks and observations.

### Selection of variables

The association of bobcat and lynx occurrences with 39 variables was evaluated across three scales, each described by a map layer (Table 1, S1 Methods). Variables were selected to describe: 1) fragmentation features such as edge, availability of cover, and patch size, 2) basic landscape features including availability of water, elevation, slope, and slope aspect, 3) density of anthropogenic features such as roads and urban areas, 4) density of habitat types and cover. Maps used to construct variable layers included the 2006 National Land Cover Database (NLCD), the National Elevation Dataset (NED), the National Hydrography Dataset, and the National Transportation Dataset. The Landscape Fragmentation Tool v2.0 for ArcMAP 9.3 [46] was used to produce fragmentation associated layers. All mapping functions were conducted at 30 m resolution in ArcMap 9.3 or ArcMap 10 (Esri, Redlands, CA).

**Table 1:** Map layers.

Scale	Local	Daily distance		Home range	Topographic		
Radius of neighborhood analysis (in meters)	60	810 Bobcat	1500 Lynx	2790	90	150	270
Agricultural (ag)–includes pasture, hay and cultivated crops	**1**	**2**	**3**	**4**			
Grasslands—grassland herbaceous, emergent herbaceous wetlands	**5**	**6**	**7**	**8**			
Coniferous forest	**9**	**10**	**11**	**12**			
Deciduous forest	**13**	**14**	**15**	**16**			
Mixed forest	**17**	**18**	**19**	**20**			
Shrub scrub	**21**	**22**	**23**	**24**			
Woody wetlands	**25**	**26**	**27**	**28**			
Developed open and low—developed open space, developed low intensity	**29**	**30**	**31**	**32**			
Developed medium and high—developed medium intensity, developed high intensity	**33**	**34**	**35**	**36**			
Forest cover—coniferous forest, deciduous forest, mixed forest	**37**	**38**	**39**	**40**			
All cover—shrub scrub, woody wetlands, coniferous forest, deciduous forest, mixed forest	**41**	**42**	**43**	**44**			
Patch—a small area of cover habitat surrounded by non-forested land cover	**45**	**46**	**47**	**48**			
Ecotone/edge—the boundary of cover within 30 meters of open habitat	**49**	**50**	**51**	**52**			
Small area of cover–< 250 acres (<1.01 km^2^)	**53**	**54**	**55**	**56**			
Medium area of cover– 250–500 acres (1.01–2.02 km^2^)	**57**	**58**	**59**	**60**			
Large area of cover–> 500 acres (>2.02 km^2^)	**61**	**62**	**63**	**64**			
Stream River edge (km/km^2^)	**65**	**66**	**67**	**68**			
Waterbody edge (km/km^2^)–streams, rivers, lakes and ponds	**69**	**70**	**71**	**72**			
Roads class1 and 2 (km/km^2^)	**73**	**74**	**75**	**76**			
Roads class 3 (km/km^2^)	**77**	**78**	**79**	**80**			
Euclidean distance to stream river edge	**81**						
Euclidean distance to waterbody edge	**82**						
Euclidean distance to cover	**83**						
Euclidean distance to class 1 and 2 roads	**84**						
Euclidean distance to class 3 roads	**85**						
Undeveloped (includes ag. areas)	**86**						
Natural habitat (excludes devel & ag.)	**87**						
Water_within100m	**88**						
Water_within150m	**89**						
Water_within300m	**90**						
Cover_within100m	**91**						
Cover_within150m	**92**						
Cover_within300m	**93**						
CoverEdge_200m –edge 100 meters inside and outside of forest (200m width total)	**94**						
CoverEdge_300m –edge 150 meters inside and outside of forest (300m width total)	**95**						
Elevation (location only)	**96**						
Slope	**97**				**98**	**99**	**100**
Aspect sin	**101**				**102**	**103**	**104**
Aspect cosine	**105**				**106**	**107**	**108**

Map number for each variable, at scales of evaluation. Raster variables were evaluated by number of pixels within the scaled neighborhood buffer, and converted to percentages for interpretation. Line densities were calculated for linear features (i.e. water and roads). Daily distance data for bobcats was taken from within an 810 m radius, and for lynx within a 1500 m radius.

The density of habitat variables was examined at three scales (local distance, daily distance [44], and female home range size) by performing neighborhood analyses on binary variable layers in ArcMap 9.3.1 [47]. A local scale with a 60 m radius was used because it is the average distance a bobcat would travel in about 15 minutes [48], and captured smaller interspersed habitat patches. The linear daily distance scale of 1.6 km diameter (810 m radius) was used for bobcats [49, 50], and 3 km for lynx (1500 m radius) [51–53]. Female bobcats in VT and lynx in ME have similar average home range sizes (22.9 km^2^ and 25.7 km^2^ respectively) [39, 43]. These were averaged to establish a home range scale covering 24.3 km^2^ (2790 m radius), under the premise that male ranges encompass female ranges [54–56].

Topographic features are localized in many areas, including the Champlain Valley, and would not have been as informative over larger neighborhoods. To provide greater definition of topography, smaller scales with 90 m, 150 m and 270 m radii were arbitrarily chosen to evaluate slope, sin aspect, and cosine aspect. Elevation data were taken at location points only.

Values for each variable (map layer) were extracted from under radio-telemetry locations. The most consistent variables within each species dataset (coefficient of variation, CV ≤ 1) were preselected and sets of uncorrelated variables were analyzed. Preselection reduced the total variables to 31 for bobcat and 35 for lynx Mahalanobis distance analyses (Table 2).

**Table 2:** Selected variables for bobcat and lynx models.

Bobcat variables				Lynx variables			
#	Name	CV	Correlations	#	Name	CV	Correlations
2	**Agricultural_810**	**0.676**		15	**Deciduous forest_1500**	**0.793**	**a**
4	**Agricultural_2790**	**0.522**	**c**^**-**^	16	**Deciduous forest_2790**	**0.606**	**a**
12	**Coniferous_2790**	**0.646**		19	**Mixed forest_1500**	**0.328**	**b**
14	**Deciduous_810**	**0.747**		20	**Mixed forest_2790**	**0.243**	**b**
16	**Deciduous_2790**	**0.530**	**d,e**	23	*Shrubscrub_1500	**0.589**	**c**
24	**Shrubscrub_2790**	**0.591**		24	**Shrubscrub_2790**	**0.521**	**c, d**^**-**^
28	**Wooded wetland_2790**	**0.589**		27	**Wooded wetlands_1500**	**0.831**	
50	**Ecotone edge_810**	**0.439**		28	**Wooded wetlands_2790**	**0.593**	
52	*Ecotone edge_2790	**0.297**		40	**All forest_2790**	**0.211**	**d**^**-**^
56	**Small Area Cover_2790 (<250 acres)**	**0.607**		41	**All cover_6**	**0.134**	**e**
30	**Development low_810**	**0.962**		43	**All cover_1500**	**0.078**	**f,g**
32	**Development low_2790**	**0.702**		44	**All cover_2790**	**0.063**	**h**
40	**All Forest cover habitats_2790**	**0.531**	**b,e**	52	**Ecotone edge_2790**	**0.579**	**i**^**-**^
41	**All cover habitats_60**	**0.645**	**a**	61	**Large area of cover_60 (> 500 acres)**	**0.219**	**e**
44	**All cover habitats_2790**	**0.446**	**b,c**^**-**^,**d**	63	**Large area of cover_1500 (> 500 acres)**	**0.097**	**f,j**
46	**Patch_810**	**0.855**		64	**Large area of cover_2790 (> 500 acres)**	**0.077**	**g,h,i** ^**-**^,**j**
48	**Patch_2790**	**0.424**		67	**Stream or river edge_1500 (km/km2)**	**0.503**	
76	**Class1 and 2 roads_2790**	**1.000**		68	**Stream or river edge_2790 (km/km2)**	**0.346**	
78	**Class3 roads_810**	**0.773**		71	**Waterbody edge_1500 (km/km2)**	**0.428**	
80	**Class3 roads_2790**	**0.447**		72	**Waterbody edge_2790 (km/km2)**	**0.300**	
85	**Distance to class 3 road**	**0.803**		79	**Class3 roads_1500 (km/km2)**	**0.760**	**k**
86	*Undeveloped_60	**0.109**		80	**Class3 roads_2790 (km/km2)**	**0.616**	**k**
87	**Natural habitat_60**	**0.588**	**a**	81	**Distance to stream or river**	**0.824**	**l**
89	**Water within 150m_60**	**0.877**		82	**Distance to waterbody edge**	**0.841**	**l**
90	*Water within 300m_60	**0.499**		84	**Distance to class 1 and 2 roads**	**0.171**	
91	**Cover within 100m_60**	**0.334**	**f**	86	**Undeveloped_60**	**0.027**	
92	**Cover within 150m_60**	**0.228**	**f**	87	*Natural habitat_60	**0.031**	
93	*Cover within 300m_60	**0.094**		91	**Cover within 100m_60**	**0.038**	
94	**Cover_edge_100mIn_100Out_60**	**0.789**	**g**	92	**Cover within 150m_60**	**0.031**	
95	*Cover_edge_150mIn_150Out_60	**0.595**	**g**	93	*Cover within 300m_60	**0.015**	
96	**Elevation**	**0.785**		96	*Elevation	**0.151**	
				97	**Slope_60**	**0.666**	**m,n,o**
	*Final model			98	**Slope_90**	**0.624**	**m,p,q**
				99	**Slope_150**	**0.559**	**n,p,r**
				100	*Slope_270	**0.473**	**o,q,r**

Selected variables for bobcat and lynx models with coefficients of variation (CV). Starred variables are contained within the final model for each species. Sets of highly correlated variables (rho>0.80) are indicated by matching letters. Negative correlations are signified by (^-^).

#### Objective 1: Principal components analysis, partitioned Mahalanobis distance analysis and mapping of movement suitability for each pixel

For each species, sets of the selected variables were analyzed using principal components analysis (PCA), which describes increasing amounts of variation within the dataset with a set of linear equations. For each PCA analysis, we were interested in the component that described the *least* amount of variation (i.e., small eigenvalues) among known locations [36], as it described variables that were most consistent across locations and considered to be minimum habitat requirements [36, 57].

For each PCA analysis, the component that described the least variation among locations provided the foundation for a partitioned *D*^2^ analysis of each location using the equation,

$$D^{2}(\text{y})=(\text{y}-\mu )’\Sigma^{-1}(\text{y}-\mu )$$

for each pixel in the study area, where y was a vector of habitat values associated with each pixel and μ was a vector of the means of these habitat variables [37]. Deviation of a point from the species mean vector was (y–μ), Σ was the variance–covariance matrix based on occupied habitat locations, and *D*^2^ was the squared distance, standardized by Σ [37]. Thus, the *D*^2^ value quantified the dissimilarity of a location from ideal (minimum) habitat with a standardized squared distance between each variable’s value at any given location (y) and the mean of each variable from known used habitat locations (μ). Low values described favorable habitat more similar to the means of the predictor variables.

Distance values describing movement habitat suitability for each location were transformed to *P*–values [37] for ease of interpretation (and, for the best PCA model, provided a basis for the connectivity analyses in Objective 2). Distance values were transformed into *P*–values for each location using:

$$P-\text{value}\;\text{for}\;D^{2}(\text{y};k)=1-\text{prob}(X^{2}{}_{\left(p+1-k\right)})$$

where the Chi Square degrees of freedom was the number of components used (*k* = 1), plus the number of variables in the model (*p*) plus 1 [37, 58, 59]. Transformation of *D*^2^ values, which have no upper limit, to *P*–values rescaled values between 0 and 1, with high *P*–values describing areas closer to ideal habitat. As habitat variables often do not meet the assumption of normality, *P*–values do not indicate probabilities [37, 59, 60].

**Fig 2:**
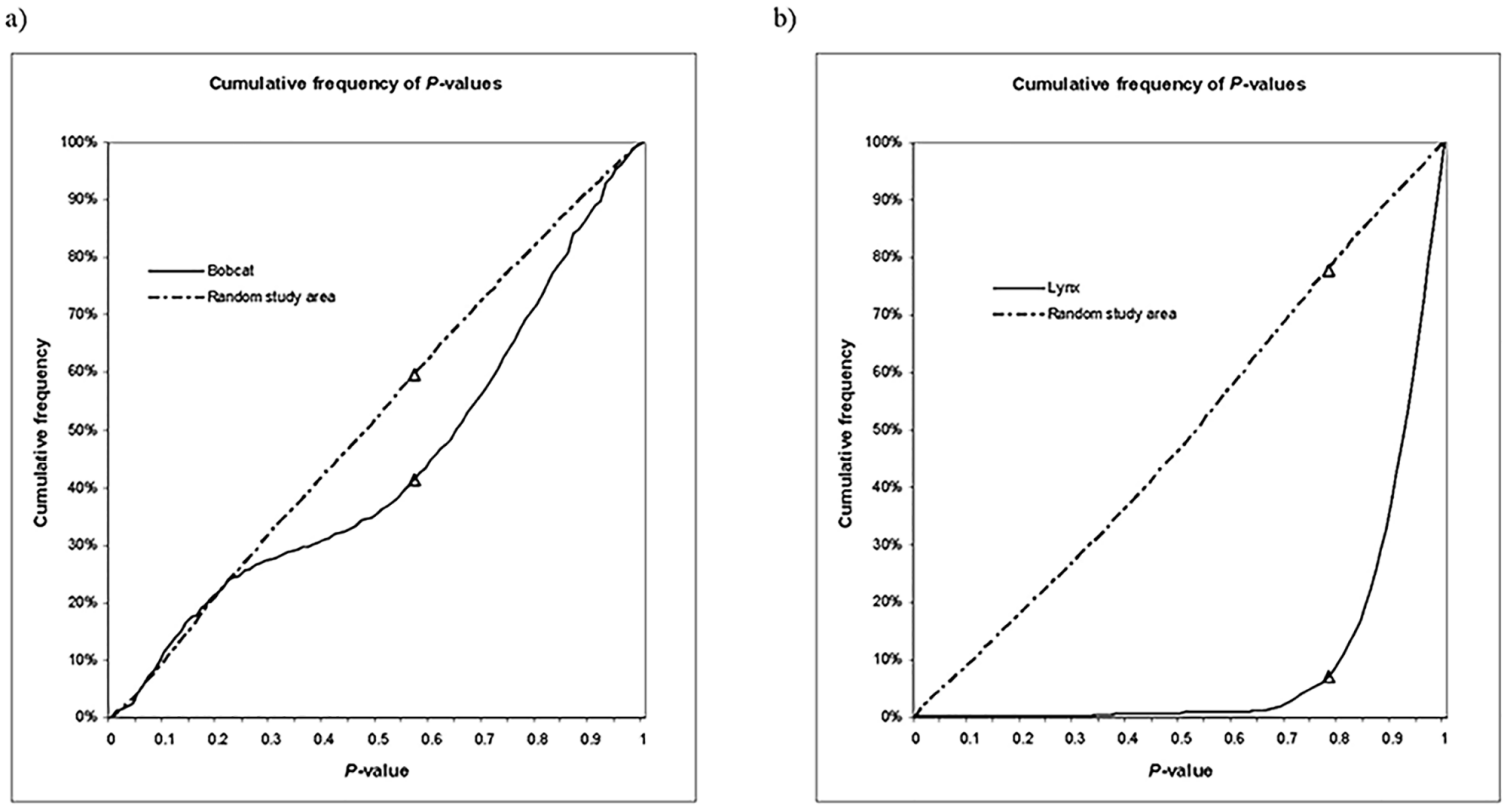
Cumulative frequency distribution curves. Cumulative frequency curve of *P*–values for bobcat (a) and lynx (b) locations, random locations within the study area, and random locations from the Northeastern region. Triangles indicate the location of maximum separation between species and random locations from each study area.

Each of the PCA models for each species was tested against random data points to identify the PCA models that provided the greatest separation between used locations and random points, and that would be used for connectivity mapping. Testing of variables against random data is common in habitat selection studies to ensure that models do not merely describe available habitat [59, 61]. Ten sets of random locations were generated within an area encompassing the bobcat or lynx radio-telemetry locations, each set equal in number to the true dataset for that species. A *P*–value was generated for each location for each candidate model, as previously described [37]. The cumulative frequencies of the 10 sets of random *P*–values were averaged in bins of 0.01 increments (*P*-values of 0.00 to 1) to produce a random cumulative frequency set of *P*–values for each candidate model. Cumulative frequencies of random and species telemetry location *P*–values were evaluated by plotting distribution curves (e.g. Fig 2). Sets of variables (models) were selected by an iterative process that disqualified variables contributing larger amounts of variation to models, and isolating variables that contributed less variation within the model and wider separation between species locations and random locations within the study area. The bobcat and lynx models that provided the greatest differentiation between locations and the landscape (random datasets) were selected for cross validation before use in connectivity analysis [59, 62].

The selected PCA models for each species were further evaluated via a leave-one-out cross validation approach, which quantified the model’s ability to predict landscape use by individual cats. Cats with few locations were merged into subsamples of > 100 data points, providing for a more robust analysis [37]. Subsamples associated with a particular animal (14 for bobcats and 19 for lynx) were withheld one at a time and the remainder of the data was used to calculate a location specific *P*–value as described previously [59]. The average *P*–value of the excluded subsample was compared to the new threshold *P*-value [63] and single sample t–tests determined which subsamples had mean *P*–values significantly below their validation threshold value.

The selected model for each species was employed to produce a predictive map across the landscape by calculating the *P*–value for each 30 m^2^ pixel across VT, NH, and ME using SAS 9.2 [37, 62]. These data described the movement suitability value of each pixel separately for each species, and formed a basis for connectivity analyses.

#### Objective 2: Identify current permeability (connectivity) across subportions of the region

We assessed the current connectivity of portions each species’ *D*^*2*^ map with Linkage Mapper v.6 [64], a program that maps cost distance linkages between habitat polygons (core areas). Subportions of VT, NH and ME (3 for bobcats and 2 for lynx) were selected for their similarity in landscape composition to the study area of each species and the likelihood they would be impacted by development. For each species, only areas with values for model variables that were similar to that species’ study area were selected. Privately conserved areas of natural habitat, state and national parks, and wildlife areas were used as habitat polygons [65], or core areas, as these are areas most likely to persist into the future. Conserved areas may include working forests. To accommodate computer capacity, polygons ≥ 75 acres (0.304 km^2^) were selected as core areas. To explore how unstudied lynx may have recently recolonized an area west of the radio-collared lynx study area, an additional connectivity map was generated using the validated lynx model and 142 buffered confirmed uncollared lynx locations from outside the study area in VT, NH and ME instead of habitat blocks.

The goal of the connectivity analysis was to identify, map, and evaluate connective habitat between polygons (core areas of conserved land) using a circuit-based least-cost corridor method to depict conductivity across the landscape (e.g., [7, 64, 66]). The initial cost (resistance) of each pixel was computed as 1—*P* from the *D*^2^ analysis. The Linkage Mapper tool (now integrated into Circuitscape), used the resistance map to compute multiple pathways between each polygon (core areas of conserved habitat) and others, and then mosaicked the results into a single composite map. The resulting map of each focal area provided an additive estimate of the relative accessibility of each cell to the nearest core area [7, 64].

We then defined “connective habitat” for each species by overlaying GPS points on the Linkage Mapper connectivity map and identifying the highest cost distance value used by each species. This maximum, rounded up by 1%, was used to set a connective habitat threshold for each species. Cost-distance pixels below this threshold were identified as “connective habitat”, while pixels exceeding this value were deemed “non-connective habitat”.

#### Objective 3: Estimate the persistence of connectivity into the future in areas most likely to experience increased anthropogenic pressure and estimate change in availability of connective habitat in these areas

The projected change in the human footprint in the Northeast [30, 67] (see also [68, 69]) was used to evaluate the impact of future development on connectivity over the next 30 years. The first human footprint analysis illustrated the relative human influence in every biome on the land’s surface [69], utilizing 9 datasets to provide a measure of human influence for each landscape based on factors such as human population density, land transformation, accessibility, and electrical power infrastructure. Forecasts of the human footprint map for the Northeast U.S. were conducted by Trombulak et al. [30], who estimated the human footprint over an approximate 30 year horizon at a 90m^2^ pixel resolution based on projected human settlement, road, and amenities over a 20 year horizon.

To determine decline in “connective habitat”, cost pixels overlapping with a projected increase in human footprint were updated by multiplying the current cost value of each pixel from the Linkage Mapper times the increase in human influence in that pixel. For example, cost pixels that occurred in locations where footprint score was projected to increase by 10% were multiplied by 1.1 to yield a projected cost. In contrast, cost pixels that occurred in locations where footprint scores were projected to decrease were adjusted downward for future projections. For example, cost pixels that occurred in locations where footprint scores were projected to decrease by 10% were divided by 1–0.1 = 0.9 to reflect increased connectivity in these areas.

The projected change in “connective habitat” from the species’ perspective in the future (the impact of projected development on connectivity) was also calculated. Each cost-distance pixel in the future map was dichotomized as being connected or not based on the threshold identified in Objective 2. We compared the change in connective habitat between the current and projected landscape for each species.

## Results

A total of 2,427 independent radio–collar GPS locations from 16 bobcats (5 F, 11 M) and 3,639 GPS collar locations from 31 adult lynx (10 F, 21 M) were used in these analyses [70]. Two young adult bobcats (B11 and B44) contributed 135 locations (S2 Table); the remaining locations were from adults. The majority (89%) of bobcat locations occurred between January and August due to collar deployment schedules [70] (S2 Fig). Lynx locations were slightly biased towards spring months, but distributed more evenly through the year [70] (S3 Fig).

### Objective 1: PCA model selection and validation and mapping of movement suitability for each pixel

Sixty eight PCA models consisting of combinations of 31 variables were considered for bobcats (Table 2). The greatest separation between cumulative frequencies for bobcat locations and random data (18.2%) was obtained at a *P*–value of 0.57 (Fig 2a) in a model including 5 variables: density of interior and exterior edges of 150 m (300 m margin) within a 60 m radius (local scale), ecotone edge density within a 2790 m radius (home range scale), and densities of undeveloped habitat (local scale), cover within 300 m (local scale), and waterbody edge within 300 m (local scale; Table 3a). A total of 58.6% of bobcat locations, and 40.4% of random locations, fell above the *P*–value threshold. The mean *P*–value of bobcat points was 0.56, while *P*–values of random points in study area averaged 0.48. The bobcat minimum habitat model was described by the last (5^th^) component of the PCA, which explained 13.5% of the variation in the dataset (Table 4). The variables with the largest (in absolute value) eigenvalue coefficients included: CoverEdge 300 m margin—60m, Cover within 300 m—60, and Water within 300m-60m were the stronger identifiers of pixels with high bobcat movement capability within the model, which also included Undeveloped habitat within 60 m, and Edge density at the landscape scale (Table 3a).

**Table 3:** Bobcat and lynx models.

a)							
**Variable**	**Eigenvector Coefficient**	**Bobcat**			**Random study area**		
		**Mean**	**SD**	**CV**	**Mean**	**SD**	**CV**
Ecotone/edge density—2790 m	0.234	0.081	0.024	0.297	0.067	0.026	0.384
Undeveloped habitat—60 m	0.28	0.972	0.106	0.109	0.927	0.208	0.225
Water within 300 m—60 m	-0.509	0.791	0.384	0.486	0.709	0.427	0.602
Cover within 300 m—60 m	-0.512	0.99	0.093	0.094	0.942	0.221	0.235
CoverEdge 300 m margin—60 m	0.588	0.7	0.417	0.595	0.484	0.467	0.965
b)							
**Variable**	**Eigenvector Coefficient**	**Lynx**			**Random study area**		
		**Mean**	**SD**	**CV**	**Mean**	**SD**	**CV**
Shrub scrub—1500 m	0.014	0.301	0.177	0.589	0.111	0.123	1.115
Natural habitat—60 m	-0.714	0.999	0.031	0.031	0.957	0.176	0.184
Cover within 300 m—60 m	0.693	0.999	0.015	0.015	0.974	0.155	0.159
Elevation—m	0.091	373.323	56.285	0.151	291.24	157.587	0.541
Slope– 270 m	-0.04	3.863	1.826	0.473	3.68	3.429	0.932

The eigenvector coefficients of the variables from the principal component comprising the bobcat model (a) and the lynx model (b). Means, standard deviations (SD) and coefficients of variation (COV) are provided for the bobcat and lynx location data as well as the averaged 10 random datasets from within each study area. The absolute value of an Eigenvector coefficient indicates the variable’s relative strength in the model for connective habitat for each species.

**Table 4:** Results of Principal components analysis.

Species	Principal component	Eigenvalue	Proportion of total variance
**Bobcat**	5	0.675	0.135
	4	0.810	0.162
	3	1.025	0.205
	2	1.055	0.211
	1	1.430	0.286
**Lynx**	5	0.259	0.050
	4	0.500	0.100
	3	0.910	0.182
	2	1.490	0.298
	1	1.860	0.372

Results of PCA on a correlation matrix of multi-scaled environmental variables assessed at radio-telemetry locations of bobcats (upper) and lynx (lower) in their respective study areas. There were 5 variables in the best model for each species, with the 5^th^ Principal Component explaining the least variation between locations.

Cross validation revealed that the bobcat model (Table 3a) adequately described 86% of the validation sets, representing 14 of the 16 cats. The mean difference in *P*–values for individual bobcats between the final model and the validation models was 0.06, suggesting that outliers had little effect on the final model. Two male bobcats had average *P*–values significantly lower than their validation model thresholds (B27 *P* = 0.52, threshold *P* = 0.61, p < 0.0001; B29 *P* = 0.51, threshold *P* = 0.57, p = 0.0024).

Eighty four PCA models consisting of combinations of 35 variables were considered for lynx (Table 2). The lynx model showed more specialization of lynx for particular habitat: 92.91% of lynx locations, but only 22.11% of random study area locations, occurred above the *P* = 0.78 threshold (Fig 2b). The mean *P*–value for lynx locations was 0.90, whereas random points in the study area had an average *P*–value of 0.52. A maximum of 70.8% separation between the cumulative frequencies of *P*–values for lynx data and random locations was found at *P* = 0.78 (Fig 2b), in a model with 5 variables: availability of Shrub scrub within a 1500 m radius (daily distance moved), Natural habitat (local scale), availability of Cover within 300 m (local scale), Elevation, and Slope within a 270 m radius (Table 3b). Lynx habitat use was described by the last (5^th^) component, which explained 4.95% of variation in the data (Table 4). The variables with the largest (in absolute value) eigenvector coefficients were Cover within 300m at the local scale, and Natural habitat at the local scale, and thus are key indicators of areas with high lynx movement capability (Table 3b).

Cross validation revealed that the lynx model (Table 3b) adequately described 84% of the 19 validation sets for the 31 cats. The mean difference in *P*–values for validation sets between the final model and the validation models was 0.08. Two adult males (L114 and L18) and a validation set of 3 females (L131, L141, L150) had average *P*–values significantly lower than validation thresholds (L114 *P* = 0.53, threshold *P* = 0.86, p < 0.00001; L18 *P* = 0.65, threshold *P* = 0.78, p < 0.0001; L131/141/150 *P* = 0.64, threshold *P* = 0.81, p < 0.0001).

### Objective 2: Map current connectivity across subportions of the region, and then evaluate current connectivity within these subportions

**Fig 3:**
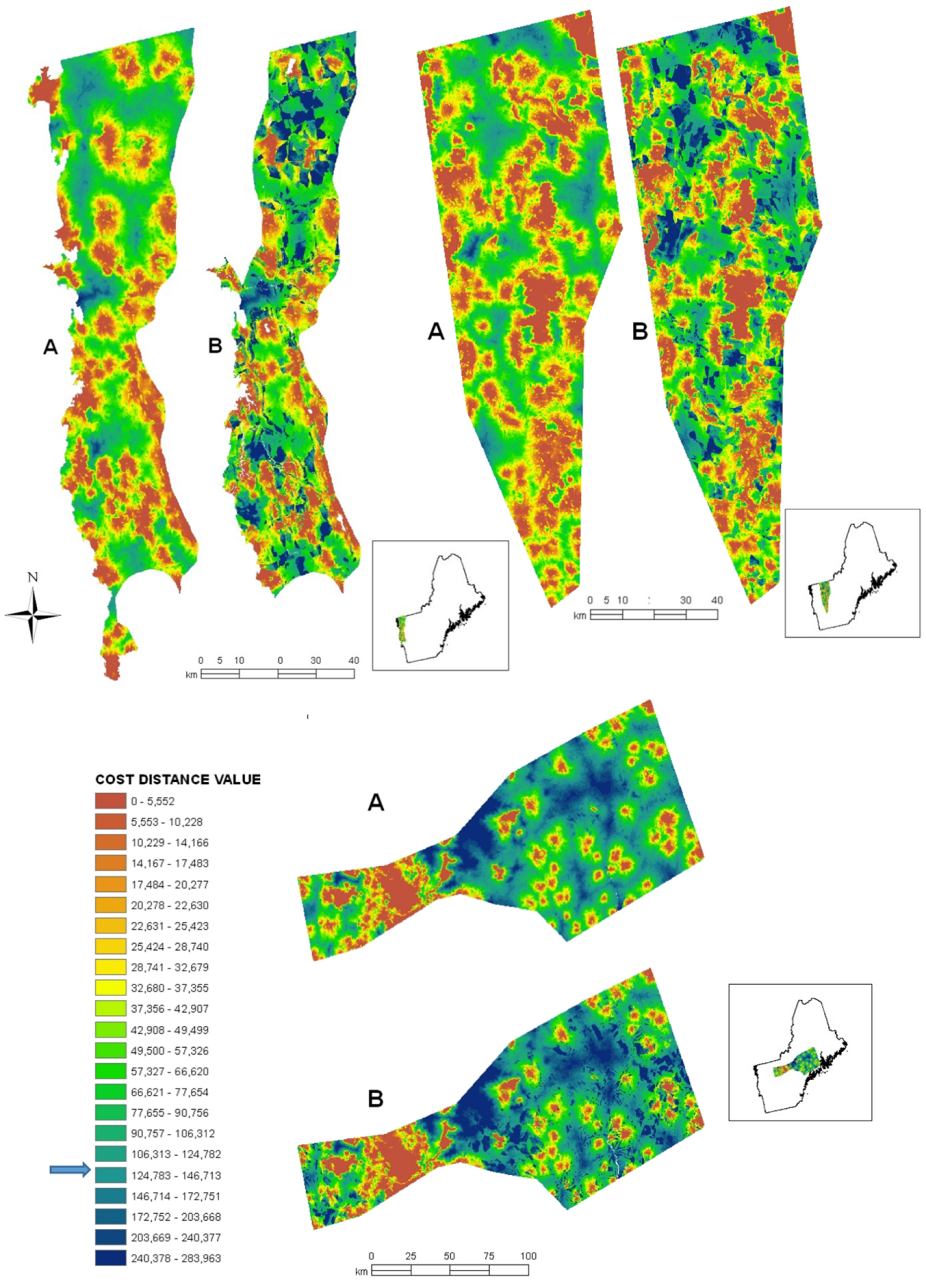
Present and predicted connectivity for bobcats. Present (a) and predicted future (b) connectivity for bobcats between conserved areas of natural habitat in the Champlain Valley Biophysical Region (upper left), the Northern Piedmont area of Vermont (upper right) and below, along the Route 2 corridor from Vermont into southern Maine (bottom). The core polygons have a cost distance of 0, and are the basis of the orange areas in the maps. Connective habitat for bobcats is defined as pixels with a cost distance value ≤ 125,000 (small arrow on legend). Future connectivity between conserved areas was predicted only where anthropogenic change has been estimated by Trombulak et al. [30].

Conserved (core) areas for bobcat comprised 202 km^2^ (4%) of the 4,879 km^2^ Champlain Valley biophysical region (CVBR), 547.81 km^2^ (8%) of the Northern Piedmont of VT, and 992.63 km^2^ (6%) of the Route 2 corridor (Table 5). Twenty percent of bobcat radio–collar locations occurred on conserved lands, with the remainder on private lands subject to future development. The highest cost distance value pixel used by bobcats was 123,523; pixels rounded up 1% to a cost distance value of ≤125,000 were defined as “connective habitat”. Connective habitat covered 3,827 km^2^ (96.5%) of the CVBR region, 6,450.67 km^2^ of the Northern Piedmont of VT (96.6%), and 11,776.93 km^2^ (69.3%) of the Route 2 corridor (Table 5, Fig 3).

**Table 5:** Conserved core areas and connective habitat.

**a)**						
Bobcat area location	Area (km^2^)	Time	Conserved Areas (km^2^)	%	Connective habitat	
					Area (km^2^)	%
Champlain Valley biophysical region	3964.9	Present	202.31	5	3827.21	96.5
	3226.7*	~2040	156.51*	5	2567.85	79.6
Northern Piedmont area of VT	6677.3	Present	547.81	8	6450.67	96.6
	-	~2040	-	-	5653.95	84.7
Route 2, VT through Augusta ME	16996.8	Present	992.63	6	11776.93	69.3
	-	~2040	-	-	10637.36	62.6
**b)**						
Lynx area location	Area (km^2^)	Time	Conserved Areas (km^2^)	%	Connective habitat	
					Area (km^2^)	%
Northeast Maine	25566.90	Present	5747.14	23	25119.86	98.3
	-	~2040			25317.65	99
Southeast Maine	23028.73	Present	2668.54	12	22618.31	98.2
	-	~2040			21833.53	94.8
Confirmed locations—VT, NH, ME	25381.85	Present	37.4**	**	25380.46***	100
	-	~2040			25299.11	99.7

Extent of conserved core areas and connective habitat within each of the areas evaluated for bobcat (a) and lynx (b), and the impact of development projected to the year 2040. Connective habitat areas, which include core habitat, are defined by the highest cost distance pixel used by each species. For bobcats this value is 123,523 (rounded to 125,000); for lynx it is 1,160,974 (1,172,610).

*As the future human footprint is not calculated for the entire Champlain Valley, the future area is smaller.

**Recent (1999–2011) buffered confirmed locations of uncollared lynx in VT, NH, and ME are used instead of conserved areas.

***The area difference of uncollared locations and present connective habitat is due to rounding error between raster and polygon layers.

**Fig 4:**
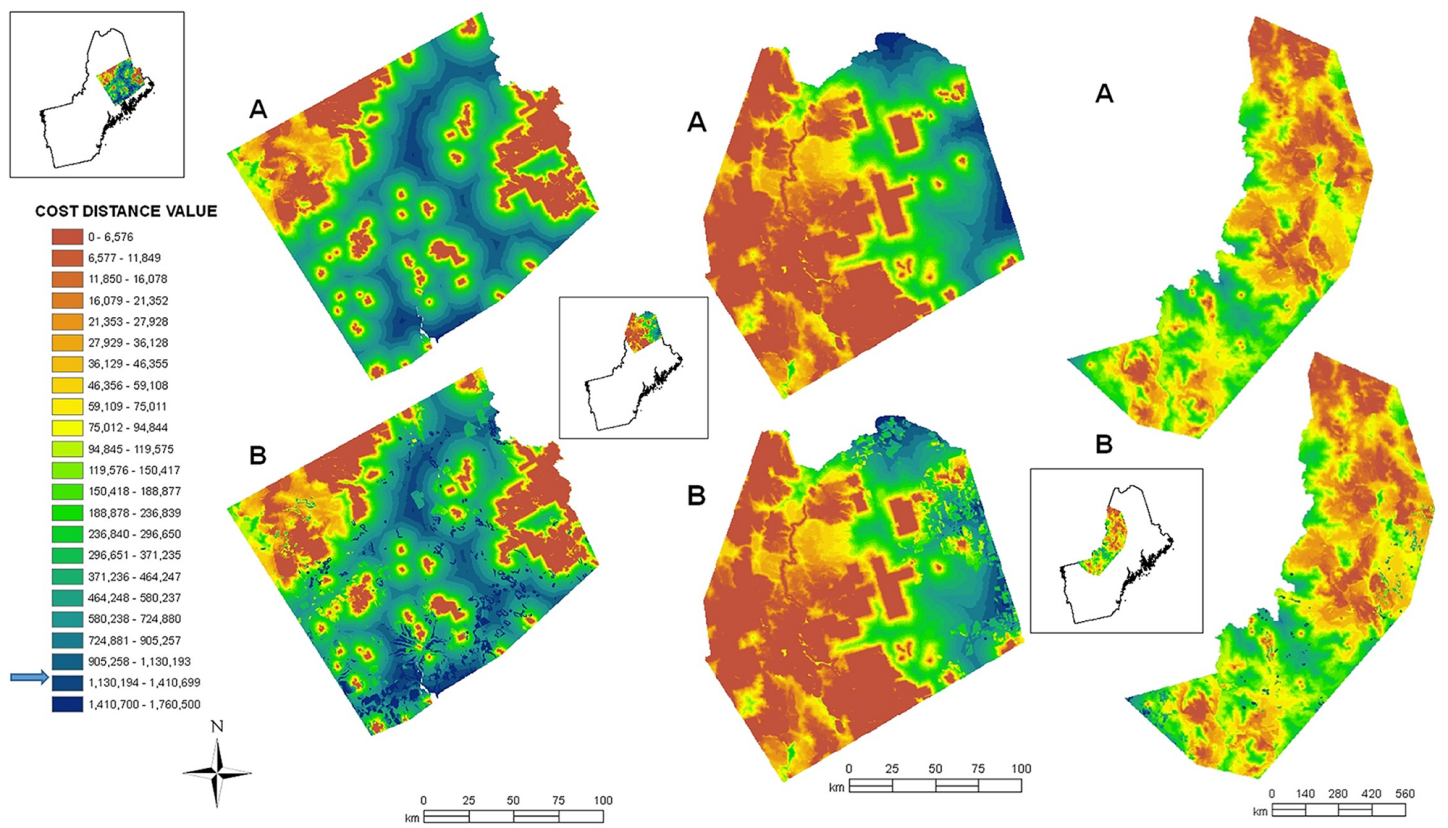
Present and predicted connectivity for lynx. Present (a) and predicted future (b) connectivity for lynx between conserved areas of natural habitat in northeastern and southeastern Maine (left and middle, respectively), and between confirmed uncollared lynx locations from ME, NH, and VT (right). The core polygons have a cost distance of 0, and are the basis of the orange areas in the maps. Connective habitat for lynx is defined as pixels with a cost distance value ≤1,172,610 (small arrow on legend). Future connectivity between conserved areas was predicted only where anthropogenic change has been estimated by Trombulak et al. [30].

Conserved areas in the lynx analyses occupied 5,747.14 km^2^ (23%) and 2,668.54 km^2^ (12%) of the northeastern and southeastern Maine areas respectively (Table 5). Only 5.4% of lynx radio–collar locations were on conserved lands. The highest cost distance value for lynx locations was 1,160,974. Rounding this up by 1% defined “connective habitat” as pixels with a cost distance value of ≤1,172,610. Present connective habitat composed 25,119.86 km^2^ (98.3%) and 22,618.31 km^2^ (98.2%) of northeastern and southeastern Maine areas, respectively (Table 5). Connective habitat covered 100% of the 25,381.85 km^2^ area surrounding confirmed locations of uncollared lynx in ME, NH, and VT (Table 5, Fig 4).

### Objective 3: Estimate the persistence of connectivity into the future in areas most likely to experience increased anthropogenic pressure

Using projections of current growth trends to approximately 2040 [30, 58], connective habitat for bobcats was estimated to decrease by 1,259.36 km^2^, (16.9%) in the CVBR, by 796.72 km^2^ (11.9%) across the Northern Piedmont area, and by 1,139.57 km^2^ (6.7%) across the Route 2 corridor (Table 5, ~ 2040 values).

Connective habitat for lynx is predicted to decrease across southeastern Maine by 784.78 km^2^ (3.4%) over the next 30 years, and across the area of uncollared lynx locations (west of the Musquacook lakes study area to Vermont) by 81.35 km^2^ (0.3%), though connective habitat was predicted to increase by 197.79 km^2^, or 0.7%, in northeastern Maine (Table 5).

## Discussion

A description of habitat use should reflect how an animal experiences its environment, which can be challenging because some species perceive habitat variables in ways that are difficult to determine [71, 72]. Other modeling methods require that variables be selected by the researcher. Our methodology allowed selection of model variables from a wide range of landscape variables at different scales to determine which best predicted occurrence of a species in an area [29, 73], based on the consistency of the variables within the data [37, 59].

Our results support previous findings that bobcats utilize a wide variety of resources [74], whereas lynx are more specific in their habitat use [75]. The separation of *P*–value cumulative frequency curves between species and random study area data illustrated that bobcats used a wider range of habitat values (available habitat) than lynx (Fig 2), as their location data had less departure from random location data (Fig 2a) than the lynx data (Fig 2b). Variables included in the final model indicated that bobcat presence is more influenced by general landscape structure and characteristics including availability of edge, cover, and riparian and undeveloped habitats than specific habitat types, which is consistent with other studies [76–78]. That two different measures and scales of edge—ecotone density at the home range scale, and density of edge at a local scale—were elements of the final model suggests that this landscape characteristic is important to bobcats. Indeed, the bobcat’s range has expanded northward as forests were fragmented, increasing edge [79, 80]. Bobcats were also found to select for edges and against interior forests at finer scales in the Champlain Valley [81], presumably in response to the combination of prey density and availability of dense cover for movement and stalking prey [82, 83]. Bobcat home range size in Mississippi was predicted by edge density; patches of cover with a large perimeter:interior ratio that provide more edge may lower search times for prey venturing outside the patch edge [78]. Lower cross validation scores for males of both species may indicate they are more likely to use suboptimal habitat in moving over wider areas, partly to access multiple female ranges [54, 84]. The two bobcats with lower validation scores were adults; further knowledge of their exact age, health and breeding status, as well as surrounding landscape changes, would have been informative.

In contrast to bobcats, which used undeveloped habitat including agricultural lands, lynx were closely associated only with natural habitats. Natural habitat and proximity to cover were essentially consistent among locations (Table 3). Lynx reliance on larger scale variables supports Fuller and Harrison [85], who found lynx select large patches of habitat with high prey density rather than areas within patches. Low use of areas with *P*–values < 0.8 (Fig 2b) suggests that this species is highly selective. Inclusion of elevation in the model supports a strong association of lynx with deeper snow found in other studies [86]. The boundary between bobcat and lynx ranges is often defined by deeper snow, where lynx might also be able to escape competition from coyotes (*Canis latran*s) [83, 87]. Climate change may reduce snow depth in the future, contracting the lynx range [88] and expanding the bobcat range.

### Connectivity

**Fig 5:**
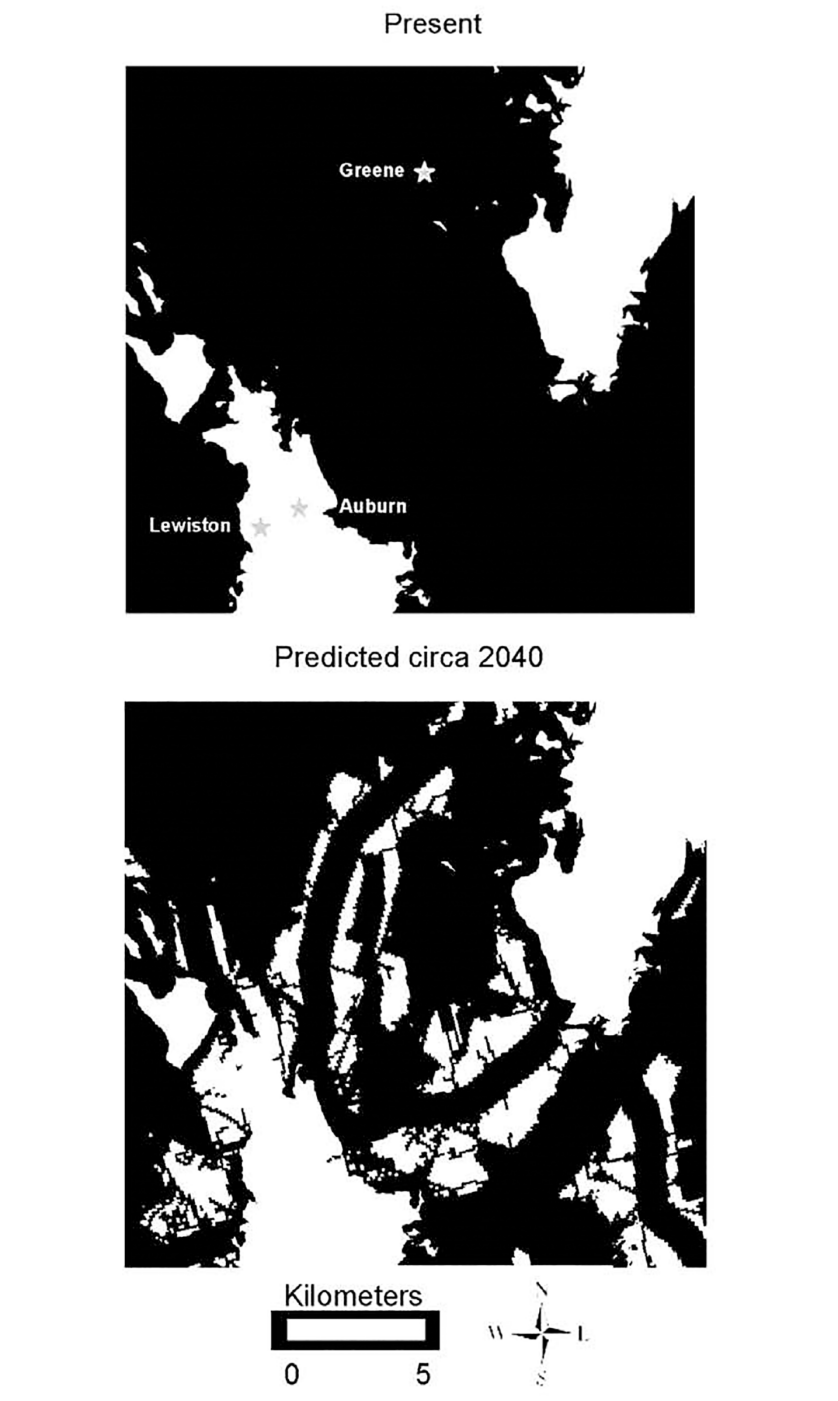
Subportion of area where connectivity is expected to decline. The impact of future development on connective habitat for bobcats in a portion of southeastern Maine around Auburn and Lewiston. Black describes areas of connective habitat, white defines pixels with a cost distance value greater than 125,000, above the maximum cost distance value used by bobcats.

Anthropogenic development is expected to have different impacts on connectivity for lynx and bobcats in the areas we studied. Mapping connective areas using circuit-based least-cost path analysis and examining how anthropogenic changes are likely to impact the landscape at a 30 m x 30 m resolution identified areas of this multiuse landscape where connectivity could decline substantially (Figs 3 and 4, Table 5). In the absence of suitable new protected areas, connective habitat for bobcats is predicted to decline in all areas studied, and movements between subpopulations of bobcats may decrease. For example, connectivity is currently largely intact across much of the eastern portion of Route 2 around Auburn and Lewiston, Maine (Fig 5). Although the Route 2 corridor as a whole is predicted to experience the smallest overall decline in connective habitat for bobcats of the three areas examined (Table 5), subportions predicted to undergo decline were concentrated, and these areas will experience restricted connectivity (Figs 5 and 3). Strategic planning and conservation of additional lands in areas expected to experience increased anthropogenic activity could minimize the impacts of development on future landscape connectivity for bobcats.

In contrast to bobcats, lynx connectivity is expected to remain stable in most areas examined. Connective habitat in southeastern Maine is predicted to undergo contraction in some areas and localized expansion in others (Fig 4), such that the impact of the increased human footprint in localized areas is not apparent when looking at numerical estimates of decline in area of connective habitat (Table 5). Although concentrated development could affect the future dispersal ability of lynx to southeast Maine, suitable habitat could also diminish with a warming climate [88]. Declines in connective habitat are expected to be minimal in areas between confirmed locations of uncollared lynx and do not appear to obstruct overall dispersal ability (Table 5; Fig 4 right), allowing continued recolonization of lynx across this portion of their former range with favorable snowdepth. Overall, connectivity is not expected to decline for lynx across their breeding range in northern New England; and an increase in connective habitat in northeastern Maine (Table 5, Fig 4) may even assist long-distance movement of animals between breeding populations in Canada and the US.

### Scope and limitations of the method

Our approach provides a conservative illustration of future connectivity, especially for bobcats, because: 1) models for each species are based on data largely from adults, who are less likely to disperse, so connectivity mapping is conservative. Only two of the animals, bobcats B11 and B44, were subadults and these cats have relatively few data points (33 and 102 respectively). Thus, the models largely reflect habitat use of individuals settled into home ranges; 2) bobcats are flexible in their habitat use, which allows them to occupy such a wide variety of habitats lower 48 United States and Mexico [71, 80]. Because our models reflect habitats found in the Northeastern US, they are best applied to this region; and 3) we used permanently conserved areas as habitat blocks, and assumed that conserved areas of natural habitat are suitable for each species. The loss of connectivity is not calibrated to the increase in human footprint projections of Trombulak et al. [30], so the time line of these predictions may vary from theirs, and periodic reevaluation is recommended.

Several caveats in terms of modeling species’ distribution are worth highlighting. First, depending on species, analysis by sex and age, and analyses of habitat use by season and time of day could be informative if the data were robust enough. Information on temporal use of habitats by species could allow schedule adjustments of landscape use by humans to also allow wildlife use. Second, although there is evidence that snow depth affects distribution of lynx and bobcats, it was not possible to correlate locations with snowdepth due to the lack of localized detail in the snowdepth data that were available. Elevation was suggested to provide some proxy for this. Third, it should be noted that connectivity is not always a gain for populations; depending on configuration, it can also facilitate the spread of emerging diseases and invasive species [89, 90].

Only areas with variable values similar to those of the study areas for each species were selected for connectivity modeling, as we assumed the models applied best to these landscapes. Though there is cohesion of individuals to the models (S4 Fig), there are subpixel landscape features, including narrow hedgerows that animals use, which are not discernable in a 30 meter pixel. Although there is no detailed confirmation of either model by dispersing animals after the data were taken, less than a year after the conclusion of the modeling and mapping, lynx were confirmed to be breeding in northeastern Vermont, in an area predicted as most favorable habitat by the 30 meter pixel suitability model.

### Conclusion

A sufficient network of connective habitat can facilitate evolutionary responses to climate change in a fragmented landscape by allowing species to adjust ranges [91, 92]. Predictions of future connectivity rely on how much of the resulting matrix of core and connective habitat persists through time. Much of the habitat in the Northeast is under private ownership, and not conserved. If additional areas are conserved, especially in pinch points identified in smaller scale views of the 30 m^2^ pixel maps (i.e. Fig 5; also see [93]), prospects for future connectivity could improve. Regular monitoring and evaluation of anthropogenic and climate induced changes on connectivity will be necessary for unbiased mapping. By considering the influence of future landscape changes [94], and adapting management as science is updated [95, 96], we can plan for and adaptively manage range adjustment by both plant and animal species [89, 97–102].

Information on the ability of wide–ranging species to negotiate a human–modified landscape can inform efforts to construct and manage functional connectivity for wildlife, helping to plan a landscape that accommodates both humans and wildlife. Behavioral studies of how focal species negotiate these landscapes spatially and temporally are integral to any models. Investigations of landscape use should be conducted at scales that are realistic to the species studied, even when connectivity is to be projected across large areas [22]. A landscape accommodating bobcat movement would include sufficient edge and undeveloped habitats. As bobcats appear to cue into landscape features at a smaller local scale, smaller portions of essential elements in a heterogeneous landscape would suffice to encourage bobcat movement between areas of core habitat. Both bobcats and lynx require access to cover. Larger areas of coniferous natural habitat, interspersed with areas of regenerating coniferous sapling or shrub scrub supporting sufficient prey, would provide connectivity for lynx [20, 70].

It is important to emphasize that habitat maps do not predict presence of a species, but rather illustrate areas a species may be able to access. Interactions with other species, human activity, human use of adjacent areas, and other variables not explored in our analyses can affect distribution. The majority of lynx and bobcat locations used were on private lands, and these areas can be subject to rapid change. Confirmed presence of breeding animals in an area from field surveys can help prioritize areas for conservation [103, 104]. We suggest that presence of target species, distribution of undeveloped areas, effects of climate change, human use of areas, and predicted development be reassessed periodically to guide efforts to maintain regional connectivity for bobcats, lynx and other wide–ranging species.

## Supporting information

S1 Methods Map layers. Construction of 108 map layers, each a variable considered in models. This supplement describes the maps in Table 1, and their construction.
(DOC)

S2 Methods Analysis overview. (DOCX)

S1 Table Bobcat location data used for analyses. (XLS)

S2 Table Capture data, and information on dates and numbers of locations used per bobcat. Data for four bobcats with large datasets was selected to even out distribution of data through the year. A star* in the Used location dates column indicates that locations from these cats were selected for months that were lacking elsewhere in the dataset. A^~^ for B4 and B11 indicates that these were young adults at the time data were gathered, and possibly dispersing.
(TIF)

S3 Table Capture data, and information on dates and numbers of locations used per lynx collar. Six lynx were recaptured and recollared and data from multiple collars used (i.e. L140 has 35+11 locations). Data was taken from the full range of dates for all lynx. All lynx providing data were adults.
(TIF)

S1 Fig Bobcat habitat *P*–values mapped by 30 m^2^ pixel across Vermont, New Hampshire and Maine, with bobcat study area indicated. (TIF)

S2 Fig Months of bobcat locations used in modeling, by cat. Dashed lines indicate that not all locations from that month were used, and that locations were selected to even out locations over the year.
(TIF)

S3 Fig Months of lynx locations used in modeling, by cat. (TIF)

S4 Fig Close up of bobcat connectivity map, with bobcat locations layered on top. (TIF)
