# Supplementary material for: Landscape connectivity for bobcat (Lynx rufus) and lynx (Lynx canadensis) in the Northeastern United States
Source: PLoS One. 2018 Mar 28;13(3):e0194243. doi: 10.1371/journal.pone.0194243 (PMC5874025; doi:10.1371/journal.pone.0194243)
Supplement: S1 Methods — Construction of 108 map layers, each a variable considered in models. This supplement describes the maps in Table 1, and their construction. (DOC) [file pone.0194243.s001.doc]

**Supporting information**

Supporting information 1. Construction of 108 map layers, each a variable considered in models. This supplement describes the maps in Table 1, and their construction.

Supporting information 1

**Map layers**

All mapping functions were conducted at 30 m resolution in ArcMap 9.3 or ArcMap 10 (Esri, Redlands, CA) using layers from the National Map Viewer (http://viewer.nationalmap.gov/viewer/). Maps used to construct variable layers included the 2006 National Land Cover Database (NLCD), the National Elevation Dataset (NED), the National Hydrography Dataset, and the National Transportation Dataset. The 2006 National Land Cover Database (NLCD) was reclassified into binary layers for each habitat type. Agricultural classes were merged (Table 1; layers 1*–*4*). Grassland/herbaceous and emergent herbaceous wetlands were merged (layers 5*–*8*). All natural habitats providing cover were evaluated separately (layers 9*–*28*), and also merged into one layer of cover habitat (layers 41*–*44*). Developed classes were merged into two layers, representing < and > 50% impervious surfaces (layers 29*–*36*). All developed areas were excluded to make a layer of undeveloped areas including agricultural lands (layer 86*). A layer of natural habitat was constructed by excluding developed and agricultural areas (layer 87*). Barren land was sparse within the study areas so was not examined. Topographic layers were derived from the National Elevation Dataset (NED; layers 96*–*108*). A layer of streams and river riparian edges was created from the National Hydrography Dataset (layers 65*–*68*); a separate layer merged riparian edges with lake and pond edges to include all water body edges (layers 69*–*7*2). Class 1 and 2 roads (federal and state highways) from the National Transportation Datasets were merged (layers 73*–*76, 84*). Class 3 roads, included local feeder and dirt roads (layers 77*–*80, 85*). Euclidean distance to cover, streams and rivers, all water bodies, and roads (classes 1and 2, and class 3) were calculated for each pixel (layers 81*–*85*); these were not scaled.

The Landscape Fragmentation Tool v2.0 for ArcMAP 9.3 (Parent and Hurd 2009) was used to produce fragmentation associated layers (layers 45–64*) from the natural cover habitat layer. Setting a narrow edge (30 m) allowing evaluation of ecotone density within each buffer. Maps exploring each species’ fidelity to interior and exterior edges of 100 m and 150 m (200 m and 300 m margins; Harper et al. 2005) were produced by merging additional habitat edge maps with distance to cover maps (layers 94–95*). Affinity for cover and water were investigated using layers 88–93*.

- *Please see Table 1 reproduced below for layer/variable names*

Parent J, Hurd J (2009) Landscape Fragmentation Tool (LFT) v2.0. Center for Land use Education and Research. University of Connecticut. Available from http://clear.uconn.edu/tools/lft/lft2/ (accessed 9 June 2011)

Map layers. Map number for each variable, and scales of evaluation. Raster variables were evaluated by number of pixels within the scaled neighborhood buffer, and converted to percentages for interpretation. Line densities were calculated for linear features (i.e. water and roads). Daily distance data for bobcats was taken from within an 810 m radius, and for lynx within a 1500 m radius. (*Map numbers are referred to above.)

| **Scale** | **Local** | **Daily distance** | | **Home range** | **Topographic** | | |
| --- | --- | --- | --- | --- | --- | --- | --- |
| **Radius of neighborhood analysis in meters** | **60** | **810** | **1500** | **2790** | **90** | **150** | **270** |
| Agricultural – includes pasture hay and cultivated crops | **1** | **2** | **3** | **4** |  |  |  |
| Grasslands – grassland herbaceous, emergent herbaceous wetlands | **5** | **6** | **7** | **8** |  |  |  |
| Coniferous forest | **9** | **10** | **11** | **12** |  |  |  |
| Deciduous forest | **13** | **14** | **15** | **16** |  |  |  |
| Mixed forest | **17** | **18** | **19** | **20** |  |  |  |
| Shrub scrub | **21** | **22** | **23** | **24** |  |  |  |
| Woody wetlands | **25** | **26** | **27** | **28** |  |  |  |
| Developed open and low – developed open space, developed low intensity | **29** | **30** | **31** | **32** |  |  |  |
| Developed medium and high – developed medium intensity, developed high intensity | **33** | **34** | **35** | **36** |  |  |  |
| Forest cover – coniferous forest, deciduous forest, mixed forest | **37** | **38** | **39** | **40** |  |  |  |
| All cover – shrub scrub, woody wetlands, coniferous forest, deciduous forest, mixed forest | **41** | **42** | **43** | **44** |  |  |  |
| Patch – a small area of cover habitat surrounded by non forested land cover | **45** | **46** | **47** | **48** |  |  |  |
| Ecotone/edge – the boundary of cover within 30 meters of open habitat | **49** | **50** | **51** | **52** |  |  |  |
| Small area of cover – < 250 acres (<1.01 km^2^) | **53** | **54** | **55** | **56** |  |  |  |
| Medium area of cover – 250*–*500 acres ( 1.01*–*2.02 km^2^) | **57** | **58** | **59** | **60** |  |  |  |
| Large area of cover – > 500 acres (>2.02 km^2^) | **61** | **62** | **63** | **64** |  |  |  |
| Stream River edge (km/km^2^) | **65** | **66** | **67** | **68** |  |  |  |
| Waterbody edge (km/km^2^) – streams, rivers, lakes and ponds | **69** | **70** | **71** | **72** |  |  |  |
| Roads class1 and 2 (km/km^2^) | **73** | **74** | **75** | **76** |  |  |  |
| Roads class 3 (km/km^2^) | **77** | **78** | **79** | **80** |  |  |  |
| Euclidean distance to stream river edge | **81** |  |  |  |  |  |  |
| Euclidean distance to waterbody edge | **82** |  |  |  |  |  |  |
| Euclidean distance to cover | **83** |  |  |  |  |  |  |
| Euclidean distance to class 1 and 2 roads | **84** |  |  |  |  |  |  |
| Euclidean distance to class 3 roads | **85** |  |  |  |  |  |  |
| Undeveloped | **86** |  |  |  |  |  |  |
| Natural habitat | **87** |  |  |  |  |  |  |
| Water_within100m | **88** |  |  |  |  |  |  |
| Water_within150m | **89** |  |  |  |  |  |  |
| Water_within300m | **90** |  |  |  |  |  |  |
| Cover_within100m | **91** |  |  |  |  |  |  |
| Cover_within150m | **92** |  |  |  |  |  |  |
| Cover_within300m | **93** |  |  |  |  |  |  |
| Edge_InOut100 – edge 100 meters inside and outside of forest (200m width total) | **94** |  |  |  |  |  |  |
| Edge_InOut150 – edge 150 meters inside and outside of forest (300m width total) | **95** |  |  |  |  |  |  |
| Elevation **96** |  |  |  |  |  |  |  |
| Slope | **97** |  |  |  | **98** | **99** | **100** |
| Aspect sin | **101** |  |  |  | **102** | **103** | **104** |
| Aspect cosine | **105** |  |  |  | **106** | **107** | **108** |
