# Supplementary material for: Landscape connectivity for bobcat (Lynx rufus) and lynx (Lynx canadensis) in the Northeastern United States
Source: PLoS One. 2018 Mar 28;13(3):e0194243. doi: 10.1371/journal.pone.0194243 (PMC5874025; doi:10.1371/journal.pone.0194243)
Supplement: S2 Methods — (DOCX) [file pone.0194243.s002.docx]

**Analysis overview**

The association of bobcat and lynx occurrences with 108 variables, each described by a

map layer, was evaluated at multiple scales. Data for each variable was extracted from

under each telemetry location and for a set of random locations taken within the study

area of each species. Variables with a coefficient of variation (COV) < 1 for species data

were preselected for modeling because our analysis was based on finding variables most

consistent within the dataset. Sets of variables were subjectively selected and each set

analyzed to find models describing the widest separation in habitat values between

species locations and random locations within the study area. Validation was used to

select models describing landscape characteristics that each species cued into most in

their environment.

Each set of variables was analyzed using a principle components analysis (PCA),

which describes decreasing amounts of variation within the dataset with a set of linear

equations (components). Each of these components/linear equations became a model. Each individual component within a PCA provided the analytical basis for an individual partition in a subsequent Mahalanobis Distance Squared analysis (D^2^) that calculated distance values for each telemetry or random location within each partition. The D^2^ measured dissimilarity of a location from ideal habitat, ideal habitat being based on the means of variables within the data. Distance values describing habitat suitability for each location were transformed to P–values (Rotenberry et al. 2006) for ease of interpretation and to provide a basis for the connectivity analyses. Cumulative frequencies of species and random location P–values were compared, and the linear equations of variables (models) providing the widest separations between these two datasets were cross validated. The selected model for each species was used to calculate a P–value for each 30 m x 30 m pixel across VT, NH, and ME, and these data were used for connectivity analyses.
