# Supplementary figures and images for: Landscape connectivity for bobcat (Lynx rufus) and lynx (Lynx canadensis) in the Northeastern United States
Source: PLoS One. 2018 Mar 28;13(3):e0194243. doi: 10.1371/journal.pone.0194243 (PMC5874025; doi:10.1371/journal.pone.0194243)

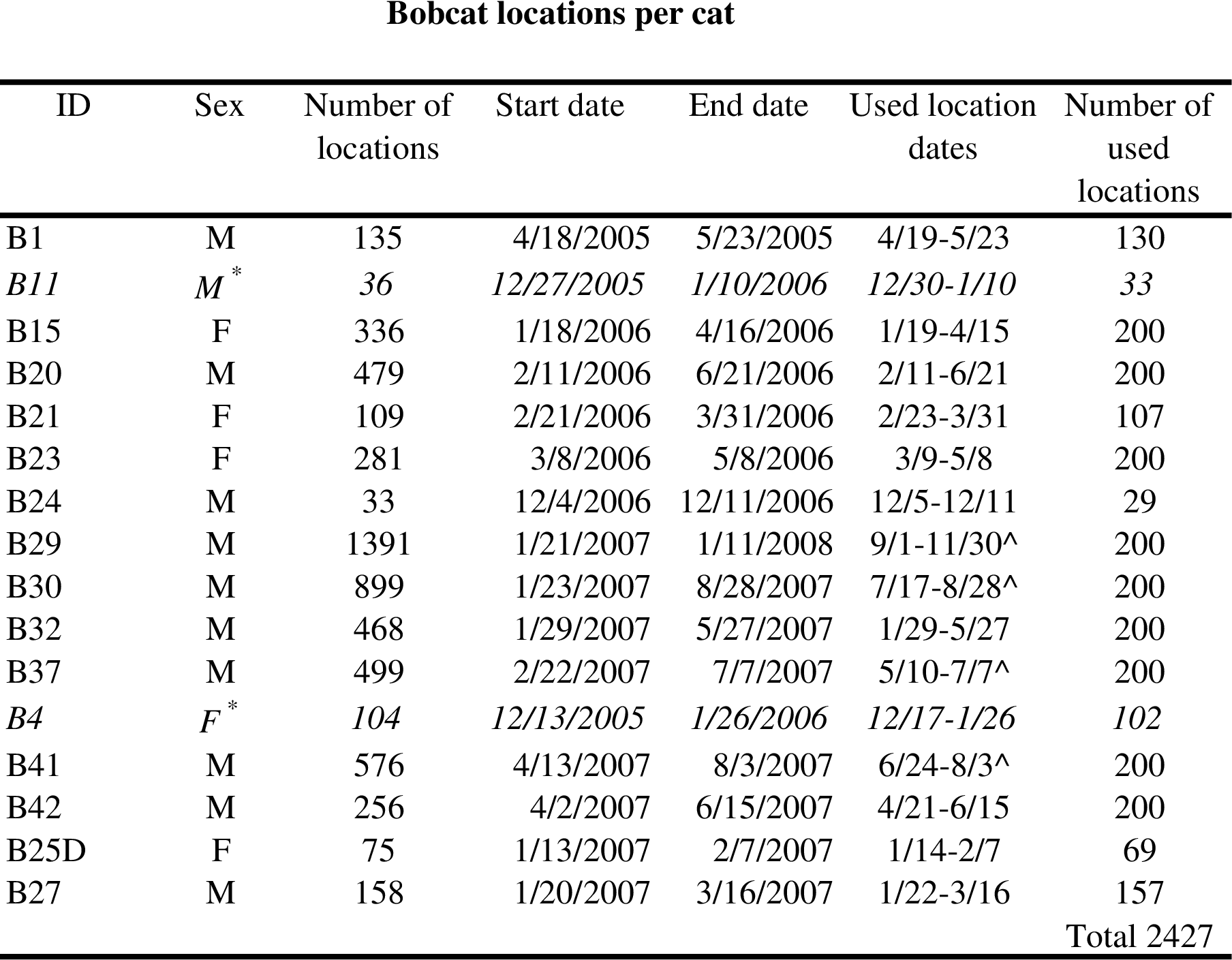

Supplement: S2 Table — Data for four bobcats with large datasets was selected to even out distribution of data through the year. A star* in the Used location dates column indicates that locations from these cats were selected for months that were lacking elsewhere in the dataset. A~ for B4 and B11 indicates that these were young adults at the time data were gathered, and possibly dispersing. (TIF) [file pone.0194243.s004.tif]

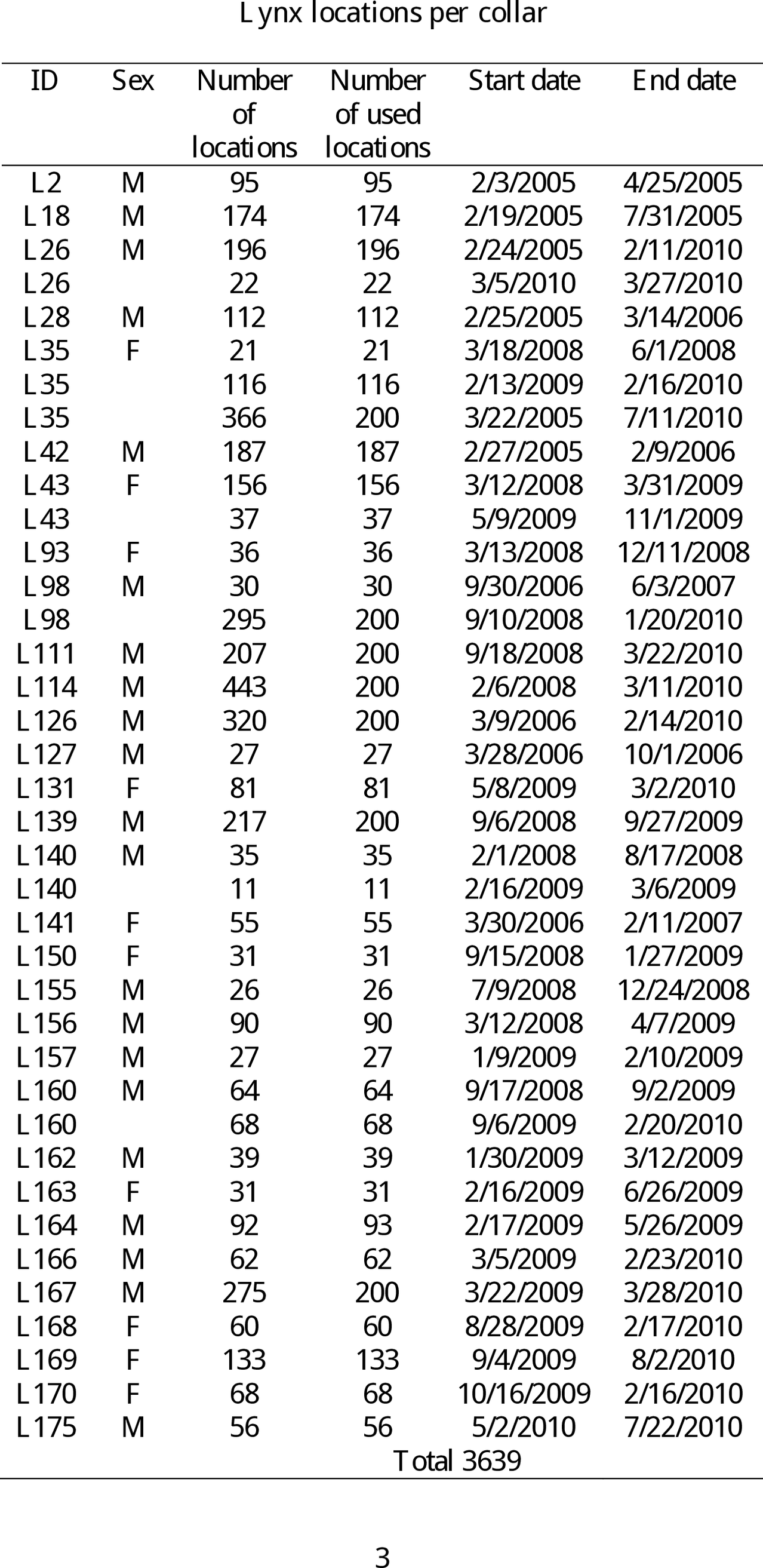

Supplement: S3 Table — Six lynx were recaptured and recollared and data from multiple collars used (i.e. L140 has 35+11 locations). Data was taken from the full range of dates for all lynx. All lynx providing data were adults. (TIF) [file pone.0194243.s005.tif]

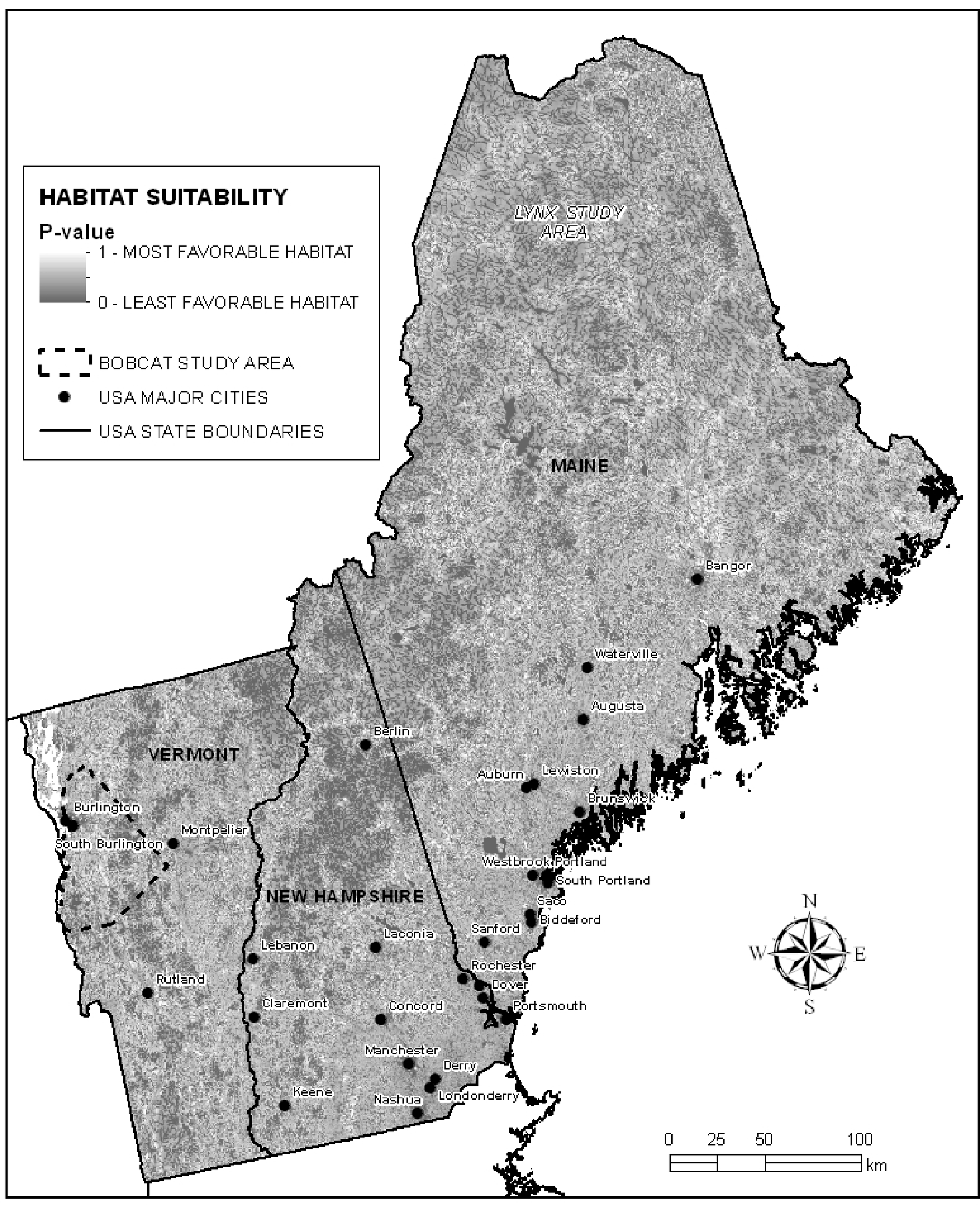

Supplement: S1 Fig — (TIF) [file pone.0194243.s006.tif]

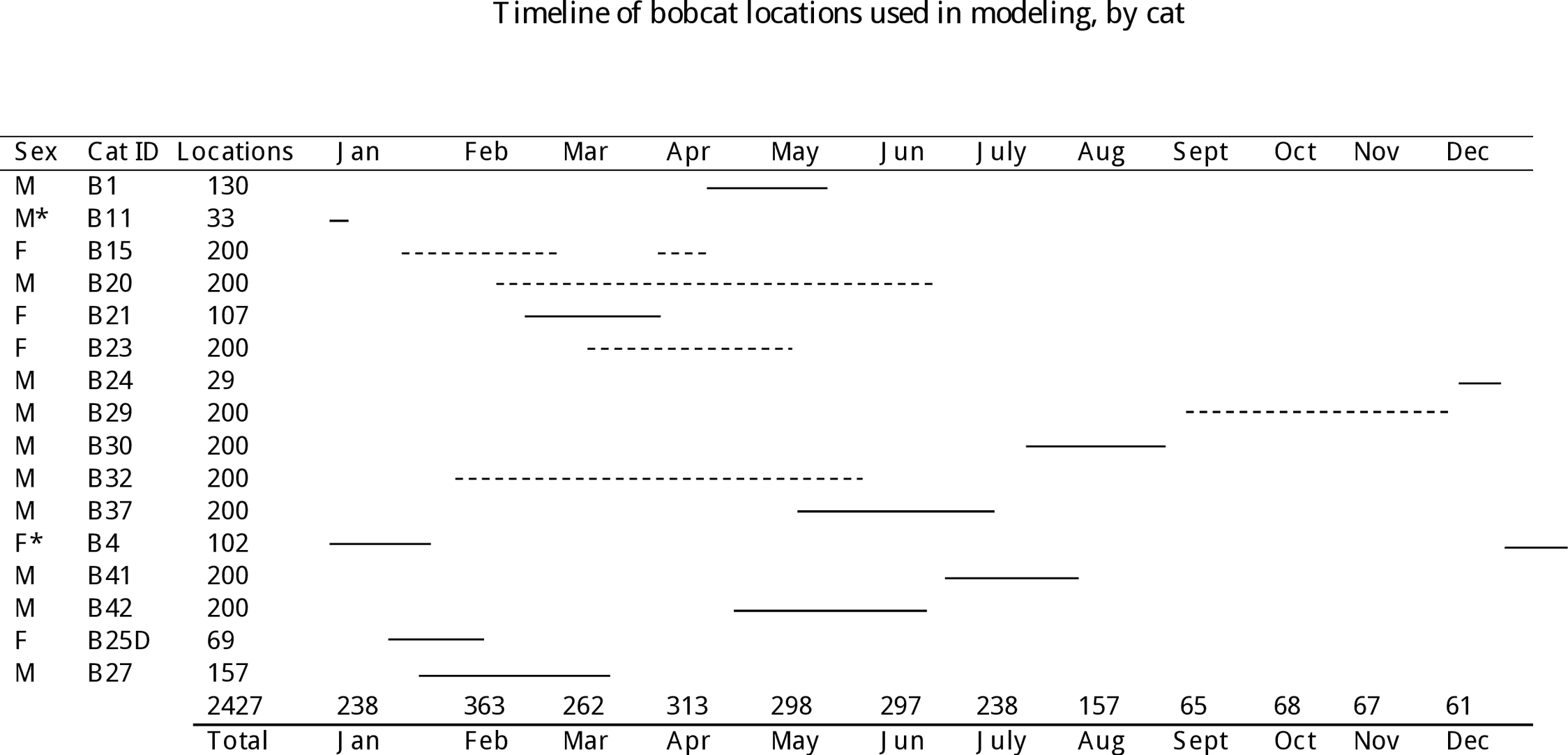

Supplement: S2 Fig — Dashed lines indicate that not all locations from that month were used, and that locations were selected to even out locations over the year. (TIF) [file pone.0194243.s007.tif]

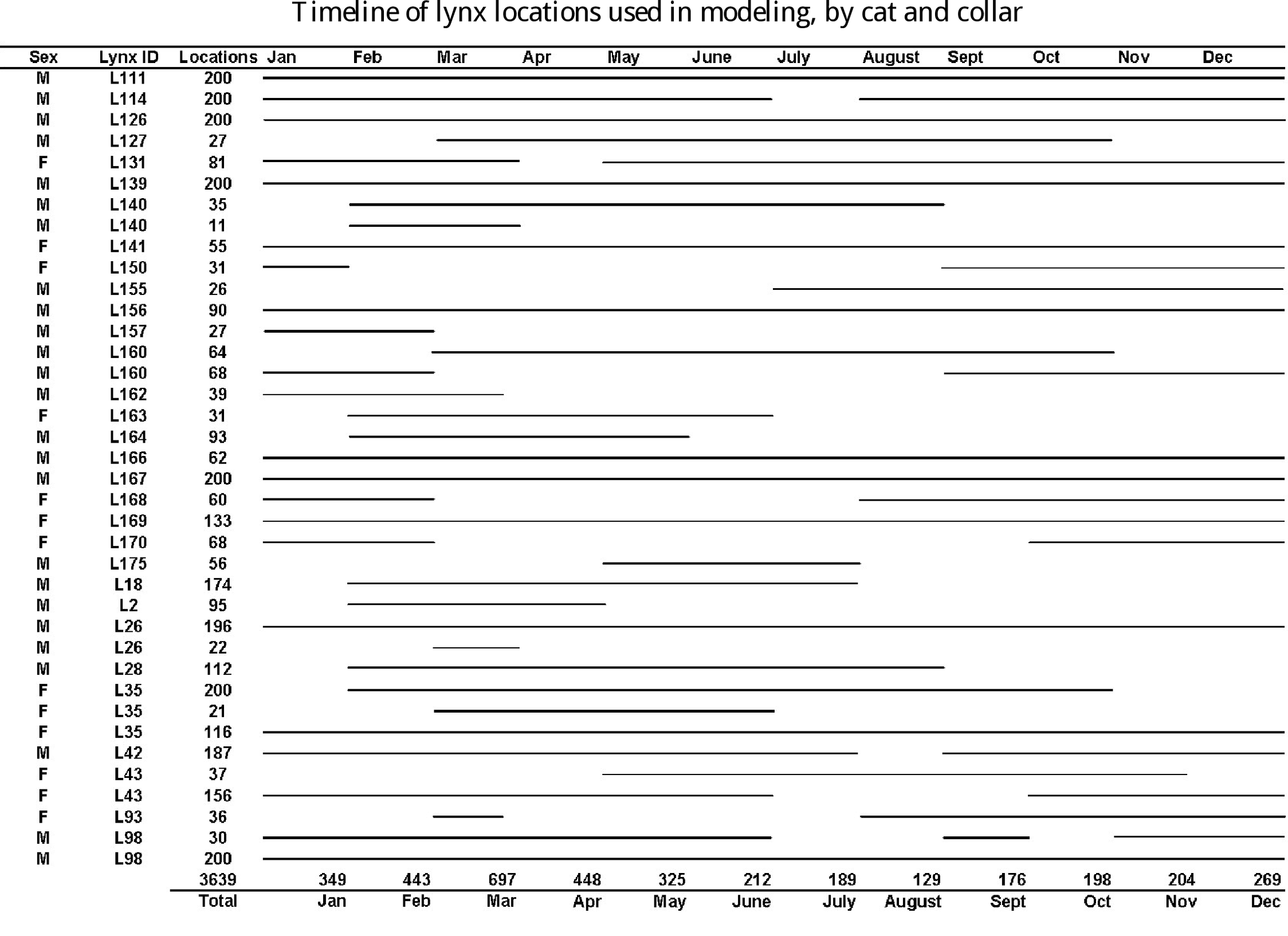

Supplement: S3 Fig — (TIF) [file pone.0194243.s008.tif]

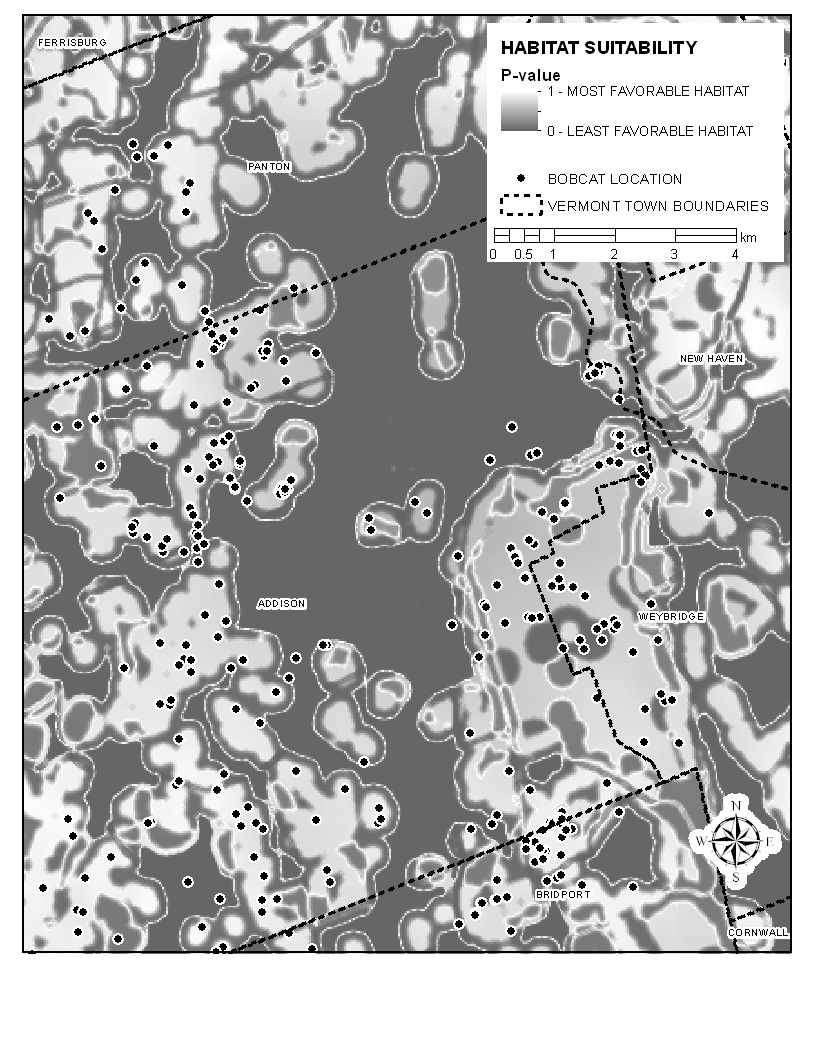

Supplement: S4 Fig — (TIF) [file pone.0194243.s009.tif]
